# Resilience Dividends and Resilience Windfalls: Narratives That Tie Disaster Resilience Co-Benefits to Long-Term Sustainability

**DOI:** 10.3390/su13084554

**Published:** 2021-04

**Authors:** Jennifer Helgeson, Cheyney O’Fallon

**Affiliations:** 1 Applied Economics Office, National Institute of Standards and Technology (NIST), Gaithersburg, MD 20899, USA; 2 Smart Grid and Cyber-Physical Systems Program Office, NIST, Gaithersburg, MD 20899, USA

**Keywords:** co-benefits, community resilience, disaster resilience, resilience dividend, resilience windfall, sustainability

## Abstract

The need for increased disaster resilience planning, especially at the community level, as well as the need to address sustainability are clear; these dual objectives have been deemed national priorities in a number of recent US Executive Orders. Major global climate agreements, (i.e., the Sendai Framework for Disaster Risk Reduction, Paris Climate Agreement, and the Sustainable Development Goals) all emphasize the need to integrate disaster resilience and climate risks with continued sustainable development concerns. Current ways of assessing synergies and trade-offs across planning for disaster resilience and sustainability in investment projects that impact communities are limited. The driving research question in this paper is how researchers and practitioners may better express relative categories of co-benefits to meet this need. We draw upon the categorization of some co-benefits as contributing to the resilience dividend, which has helped communication across fields and created bridges from research to practical on-the-ground planning in recent years. Furthermore, we leverage the growing focus on the need to recognize the role of narratives in driving decisions about how and where to invest, which elucidates the inherent value of archetypes that resonate across stakeholders and disciplines to describe investments that may meet multiple objectives. We introduce the concept of a resilience windfall as an unexpected or sudden gain or advantage of resilience planning to be conceptualized alongside resilience dividends. We then assess the practicality of decerning resilience windfalls across various projects that have aspects of both resilience and sustainability. We recount five narrative vignettes that demonstrate disaster resilience interventions and associated resilience dividends and windfalls. This effort highlights the importance of considering resilience dividends and resilience windfalls during the planning, execution, and evaluation phases of disaster resilience projects. These typologies provide an important contribution to the integration agenda between disaster resilience, climate risks, and sustainable development. There are policy implications of framing incentives for interventions that address both disaster resilience and long-term sustainability objectives as well as encouraging robust tracking of both resilience dividends and windfalls.

## 1. Introduction

In 2020, there were 22 weather/climate disaster events, each with losses exceeding $1 billion in the US [1]. The warmest decade on record was 2011–2020, and 2020 marked five straight years of exceptionally warm weather [2]. Thousands of lives were lost to extreme weather globally in 2020, with 262 deaths in the US directly attributable to natural disasters and other extreme weather events [1]. Economic effects on the areas impacted directly and indirectly were significant; Swiss Re estimated insured losses to be $83 billion, making 2020 the fifth costliest year on record since 1970 [3]. Furthermore, despite lockdowns associated with the COVID-19 pandemic, which decreased the pace of carbon contributions, all-time atmospheric records were set with a seasonal peak of 417.1 ppm in May 2020 reported (Measured at Mauna Loa Observatory, HI, USA), an indication of continued anthropogenic contributions to climate change [4].

Communities clearly need to increase their disaster resilience planning efforts, while also addressing sustainability. These dual objectives have been deemed a US National Priority [5] p. 7619. The reality of climate change as a threat multiplier is increasingly apparent [6]. Thus, the ways we assess synergies and trade-offs across planning for disaster resilience and sustainability in investment projects that impact communities needs to be reconsidered and new approaches to expressing relative categories of co-benefits need to be developed. Co-benefits refer to “effects that a policy or measure aimed at one objective might have on other objectives, irrespective of the net effect on overall social welfare” [7] p. 14. This concept offers a way to not compromise on economic growth while allowing long-term sustainability to be accounted for across decision domains.

In recent years, building designers and community planners, among others, have recognized the importance of taking an integrated approach to sustainability and resilience, but in practical application this dual objective approach is not often intentionally realized. For some time, the two efforts have been largely disconnected despite overlapping agendas and shared co-benefits. Formally integrating the two concepts in future economic evaluations is important to ensure the development of long-term sustainable infrastructure systems that can withstand increasingly frequent and intense large-scale disaster events. In particular, consideration of co-benefits is a promising way to address this issue.

When the benefits of an investment are primarily tied to losses avoided in the event of a severe hazard, the absence of a disaster event over a planning horizon can make a priori prudent investments in resilience appear unnecessary. Recent work in the field of community resilience economics investigates the co-benefits of resilience enhancing investments (e.g., [8–10]) which often address competing objectives, especially sustainability goals. Much of the work done in quantifying co-benefits of resilience planning is focused on developing and transition economies (e.g., [8]) and there continues to be a call to better address quantification of such co-benefits [11]. Benefit–cost analysis (BCA) valuation of community resilience alternatives increasingly encourages incorporation of co-benefits (see [12,13]).

Mayrhofer and Gupta [14] provide a thorough review of the use of co-benefits and note that the “co-benefits concept implies a ‘win–win’ strategy to address two or more goals with a single policy measure;” however, they concluded that the de facto potential of the concept has been marred by the fact that little transdisciplinary work had been done to explore the applications of co-benefits to political and institutional decision-making outside of attempts to apply the concept in economic analyses. In recent years, categorizing some co-benefits as contributing to the *resilience dividend* has helped communication across fields and to bridge from research to practical on-the-ground applications and planning. The resilience dividend is a term used to describe the net co-benefit (or co-cost) of investing in enhanced resilience, in the absence of a disruptive incident [11]. Terminology that resonates across groups, such as *dividend*—a token reward paid to the shareholders for their investment in a company’s equity, and it usually originates from the company’s net profits—helps capture interest and effectively and efficiently communicates the need and benefit of considering co-benefits that contribute to resilience. Rodin [15] compiled narratives elucidating cases of resilience dividends focused on community sustainability across social, environmental, and engineering perspectives.

Events over the last couple of decades (pre-COVID-19 pandemic) have left economists increasingly addressing economic resilience—which, in essence, alludes to the durability of an economy to negative shocks [16,17]. However, economic resilience is not only the ability of an economy to withstand or recover from an economic shock, but it encompasses the ability to adapt to new circumstances, including longer-term incremental changes that are recognized primarily in social and institutional functions. Over the longer-term, it implies a capacity for transformation.

In parallel, growing focus on the need to recognize the role of narratives in driving decisions about how and where to invest [18] elucidates the inherent value of archetypes that resonate across stakeholders and disciplines to describe investments that may meet multiple objectives. Rodin [15] and others have used narratives to elucidate the concept of a resilience dividend, which in turn has made the term accessible across disciplines and meaningful in the realm of policy-making. We suggest that other formulations of co-benefits should be elucidated through narrative discourse and in turn, be included in analyses of resilience and sustainable planning. Presenting information within a narrative structure engages audiences, and provides a structure for linking information, people, actions, and consequences. It also provides a larger frame for compelling messages across communities and disaster types.

In this paper we review the use of co-benefits and the resilience dividend as concepts to help motivate a business case for projects that address resilient solutions that also provide long-term sustainability. In addition, we introduce the concept of a *resilience windfall*, as an unexpected or sudden gain or advantage of resilience planning, which should be conceptualized alongside resilience dividends in community-level resilience planning and evaluation of resilience interventions. As we propose it, a net resilience windfall may derive from two sources: (1) Regular, ongoing investments that lead to a distinct additional performance improvement when the system is confronted by the disruptive objective event and/or (2) Investments that boost the system’s ability to address the onset of additional disaster events (i.e., shocks), opposed to improving (only) day-to-day operations (stressors). These concepts are explored through the use of five demonstrative narrative vignettes that focus on resilience planning, but have identifiable resilience dividends and windfalls.

Investments in resilience are generally allocated between mitigation and adaptation efforts. These investments create the resources and capabilities, that if effectively harnessed by communities, pay dividends and improve the odds of collecting windfalls. The development of mitigation measures and adaptive capacity is a fundamental challenge and opportunity for the political and economic institutions serving communities everywhere. While production functions are as diverse as the institutions and conditions found across communities, the cultivation of resilience dividends appears intensive in mitigation measures, and windfalls are most likely to be earned when investments in adaptive capacity prove especially effective.

Taken together, the concepts of the resilience dividend and resilience windfall stand to improve measures of sustainability-related resilience that can be effectively communicated for decision-making.

## 2. Background: Sustainably Resilient?

The world increasingly exhibits characteristics of a VUCA state (volatile, uncertain, complex and ambiguous) (e.g., [19]). The VUCA state is defined by an increasing likelihood of complex events arising from a combination of shocks and stressors that may arise through a complex combination of both natural and human-made causes, such as climate change, increasing occurrence and intensity of extreme weather events (EWEs), resource scarcity, increasing socio-economic inequalities, and pandemics.

With the increase in complex events there is an urgency to consider new risk management strategies, coupled with innovative ways to value synergies and trade-offs between resilience and sustainability. Synergies are the interactions between resilience and sustainability, such that their combined effects are greater than the sum of their effects if implemented separately. Trade-offs are a balancing of resilience and sustainability when it is not possible to execute on both fully and at the same time (due to financial or other constraints).

There needs to be a deeper understanding of how communities can design resilience planning efforts to complement other community-level objectives, and evaluate the potential trade-offs between investing in resilience, sustainability, and other objectives. Resilience planning and investment decisions will prove suboptimal should these complementarities and trade-offs be omitted from the evaluation of a community’s long-term sustainability strategy. These investment choices are essentially *options* that help communities manage uncertainty over future conditions. It is critical that investment of resources be used in a manner that addresses multiple objectives when at all possible. These objectives may be quite diverse, as noted above, and are often place based in nature.

### 2.1. Disturbance Types and Risk Management

In the face of complexity and turbulence, disruptions are often unforeseen and traditional risk-based practices may be inadequate. The World Economic Forum (WEF) publishes an annual report on risk factors that may hinder global economic development, ranging from climate change to technological failures to political unrest. In recent years, the report has shifted from quantifying specific risk factors to portraying the interdependencies among these factors [20]. This shift highlights the need for a probabilistic systems approach. However, developing valuation estimates ex ante for resilience planning tend to be static (e.g., benefit–cost analyses).

Complex events can result from multiple hazards, often through a multifarious combination of natural (e.g., hurricanes), biological (e.g., pandemic), and/or human-made (e.g., terrorism) causes. Pescaroli and Alexander [21] propose a holistic framework that highlights the complementarities of four risk types (compound, interacting, interconnected and cascading risks). Chronic events are recurring and often can be expected, such as the annual influenza season. Acute events are associated with less predictable hazard events that generally occur infrequently. Covariate events directly affect entities in a given geographic region, while idiosyncratic events affect specific entities within a community. Though covariate events may be experienced broadly by a community, they may still be highly localized (e.g., depth of flooding at a given property).

The need to address complex events is demonstrated by recent US Executive Order No. 14008 [5] p. 7619: “…we face a climate crisis that threatens our people and communities, public health and economy, and, starkly, our ability to live on planet Earth. Despite the peril that is already evident, there is promise in the solutions—opportunities to create well-paying union jobs to build a modern and sustainable infrastructure, deliver an equitable, clean energy future, and put the United States on a path to achieve net-zero emissions, economy-wide, by no later than 2050.” Both disaster resilience and long-term sustainability are critical to achieving these goals.

Infrastructure designed with adaptability and flexibility in mind creates options—not in the lay sense, but rather in the sense of a stock option—where an extra amount is paid now (equivalent to a premium) in return for having the potential, but not the obligation, to adapt the operation of infrastructure in the future should future conditions require it. The value of this adaptability and flexibility can be shown to increase in line with the level of future uncertainty and may be accounted for by co-benefits to resilience planning at a single period in time.

### 2.2. Resilience

Resilience is a fundamental attribute of built infrastructure that supports living systems, enabling them to resist disorder and thrive in an ever-changing world. Presidential Policy Directive 8 (PPD-8) [22] defines resilience as “the ability to adapt to changing conditions and withstand and rapidly recover from disruption due to emergencies” and Presidential Policy Directive 21 (PPD-21) on Critical Infrastructure Security and Resilience [23] expands the definition to include the “ability to prepare for and adapt to changing conditions and to withstand and recover rapidly from disruptions.” Resilience is the ability of a system to react and adapt to abrupt shocks (internal and external) and to avoid gradually occurring stressors. As systems grow larger and more structured, their resilience can wane, making them increasingly vulnerable to external disruptions and internal decay. Designing for resilience involves embracing variability rather than struggling to maintain constancy.

### 2.3. Sustainbility

Sustainability is the practice of reducing or eliminating environmental impacts. Defined broadly, long-term sustainability addresses social-ecological systems and protection of the natural environment, human, and ecological health, while driving innovation and maintaining quality of life [24]. In contrast, resilience is the practice of designing systems to endure physical, social, and economic shocks and stresses explicitly. Sustainability is not a reachable end-state, rather, it is a characteristic of a dynamic, evolving system. Long-term sustainability results not from movement along a smooth trajectory, but rather from continuous adaptation to changing conditions, which involve both stressors and shocks [25]. Sustainability in the context of climate change supports decisions that integrate both adaptation and mitigation considerations.

Even during 2020, a year dominated by adapting practices to a global pandemic, sustainability has accelerated and has expanded to include a wider range of environmental and social issues [26]. In the last five years many global goods and service categories have been disrupted by sustainability trends, including but not limited to energy, protein, transportation, and investing [26]. It is unclear whether this is spurred by concerns for individual health, carbon emissions, or animal welfare, but there are implications for land planning and subsequently resilience planning across natural disaster types (e.g., wildfire or flooding).

### 2.4. Sustainably Resilient: Synergies and Trade-Offs

Efforts to build system resilience focus on preparation for, mitigation of, and response to events such as earthquakes, hurricanes, fires, and floods; it also considers how systems need to adapt as characteristics of these hazards change over time. Sustainability is a characteristic of a dynamic, evolving system and focuses on the ability of a system to meet present and future needs. Long-term sustainability will result not from movement along a smooth trajectory, but rather from continuous adaptation to changing conditions. Design and planning for resilience can lead to synergies that help to bolster sustainability. This is because co-benefits of resilience planning often result in benefits that accrue daily, which may contribute to long-term sustainability. Yet, efforts to increase disaster resilience may also be associated with trade-offs that reduce sustainability.

Marchese et al. [27] identify three generalized frameworks for organizing resilience and sustainability that dominate in the literature: (1) resilience as a component of sustainability, (2) sustainability as a component of resilience, and (3) resilience and sustainability as separate objectives. This paper looks at a specific case of the third category, namely resilience and sustainability as concepts with separate objectives that can complement (or compete) with each other; this type of approach is like that taken by Bocchini et al. [28]. Typically, there are temporal- and spatial-scale differences with resilience achievable at a single temporal and spatial scale (e.g., [29,30]). Meanwhile, sustainability is focused upon longer temporal scales and greater spatial scales [31].

In the US, community leaders, local governments, and tribal governments bear the primary responsibility of ensuring their communities plan for and mitigate the risks posed by natural hazards and climate change impacts, respond to the effects of hazard events, and establish priorities for and manage the activities of disaster recovery after an event [32,33]. Building on the notion that “people sustain what they value,” Tainter [34] illuminates the fundamental tension between the dual community goals of sustainability and resilience: “Sustainability is the capacity to continue a desired condition or process, social or ecological. Resiliency is the ability of a system to adjust its configuration and function under disturbance.”

Communities can coalesce around shared values and common economic or political institutions. “Human institutions are always, in part, problem-solving systems, and the major problem they face is sustaining themselves” [34]. Self-preservation is the most basic of shared values possessed by communities. Paradoxically, community preservation may require action that changes what the community values, and therefore, the composition of the community itself. Citizens who value sustainability might move away from communities that seek resilience at too great a cost to sustainability. Alternatively, communities that clearly value efforts to meet both sustainability and resilience objectives will attract like minds. Perhaps the most definitive aspect of a community is how it maintains the capacity for change that is required for disaster resilience and long-term sustainability.

The ability to adjust configuration in service of problem solving may require changes in the shared values that define a community. Yet, many communities that clearly value and commit to the ability to change for the better, do so without compromising on the other core values that unite its members. At the center of the fundamental tension between resilience and sustainability is the matter of a community’s success in deconflicting of problem-solving efforts with respect to each goal. Coherent planning on dimensions of resilience and sustainability can mitigate the tendency for complexity to become a hindrance to our coexistent goals.

Co-benefits of resilience and sustainability measures related to human health often include reductions in injuries, disease, and mortality. Improvements to air quality are a common co-benefit of resilience activities. For example, many emission sources for greenhouse gases (GHGs) also emit air pollutants, such as sulfides, oxides, and particulate matter, which also have negative health impacts through aggravating respiratory illnesses. Resilience and sustainability activities that limit GHG emissions or impose caps on emissions are also likely to reduce other harmful emissions and associated impacts as a co-benefit. A resilience project to mitigate wildfire spread contains another example of a co-benefit of improved air quality, as a decrease in wildfire spread or intensity would result in less smoke and comparative increases in air quality. Communities may be more apt to invest in resilience planning if it is perceived that co-benefits reduce the opportunity costs of investment to address other objectives.

### 2.5. Co-Benefit Narratives

Communities may be geographically defined by boundaries and a leadership structure; however, a community’s values are defined by its shared experiences and the narratives that emerge from it. Shiller [18] notes that “narratives have the ability to produce social norms that partially govern our activities, including our economic actions.” Attention is scarce, and humans often find stories more compelling than statistics. For many, resilience and sustainability are nebulous concepts until a focusing event, such as a major disaster converts the ephemeral but shared experiences of community members into a motive force for collective change. As community members evolve their views regarding the threats they face, focusing events can change what a community values and how it invests. Therefore, what a community values and how it approaches sustainability and resilience is susceptible to path dependence and a bias towards solving the problems that dominate popular narratives.

Communities across the world confront similar challenges surrounding disaster resilience and sustainability planning; the opportunity for these dispersed groups of people to learn from one another is substantial, and could be aided by high quality, compelling and scientifically sound narratives. Given that narratives continue to emerge from human experience as a matter of course, using these to better structure collective understanding of the possibilities surrounding relative co-benefits is appropriate. Let us not ignore the findings that, “narratives are easier to comprehend, and audiences find them more engaging than traditional logical-scientific communication” [35]. The use of narrative expressions “provide important, but underutilized insights for understanding affordances and obstacles to collective behavior change” towards sustainability for communities [36]. Furthermore, narrative expressions can be harnessed to influence transitions to more sustainable collective behaviors and improve community responses to change in a VUCA world [37]. Valuation and documentation of dividends and windfalls can contribute to why a narrative about a resilience investment resonates with others and why it is worth sharing.

Quoting a motion picture executive on the challenge of predicting hits and flops at the box office, Shiller [18] points out that what makes an idea or story go viral is a very difficult thing to determine in advance. Those researchers interested in the subject of what determines virality or the propensity of people to share content have looked at the context of popular media such as newspaper articles [38], and academic literature in the social and physical sciences [39]. While the nuanced subject of what induces people to share narratives needs more research, we can learn from some of the empirical regularities uncovered so far. Within the context of scientific literature, “[w]hen describing one’s work to a lay audience, framing findings in a way that (i) arouses emotion or makes the work seem more (ii) useful or (iii) interesting should increase the likelihood they are shared” [39]. We view these findings to be instructive in our framing of resilience dividends and windfalls.

## 3. The Case for Co-Benefit Recognition

A precise definition of co-benefits is necessary for identification, classification, measurement, and meaningful narrative expressions to arise that link disaster resilience and sustainability. In this section we review the methods employed in this paper, the definition of co-benefits, the uncertainty under which they typically accrue, and their potential roles as option values to help communities address resilience and sustainability goals.

### 3.1. Methods and Materials

The research methodology employed herein is based on analytical efforts to categorize and extend the recognition of co-benefits under three types of resilience dividends and two types of resilience windfalls; see Sections 4.1 and 4.2. A thorough literature review on the use of co-benefits in resilience project evaluation at the community level was conducted and is described throughout our formulation of the proposed framework for resilience dividends and windfalls. Furthermore, the discussion of considering return-on-investment (ROI) implications of allowing for the option value of dividends and windfalls is practical considering typical funding streams that may be completely or partially publicly funded; see Section 4.3. The methods employed provide a framework relevant to academics and practitioners. The five narrative vignettes analyzed to determine the existence of resilience dividends and windfalls were selected after a review of grants provided over the last ten years under the Hazard Mitigation Grant Program administered by the US Federal Emergency Management Agency [33] and additional community-level disaster resilience projects. Three examples were selected for resilience interventions at a community level that had been enacted in part or entirely. These illustrative narrative vignettes were selected in order to test the proposed resilience dividend and windfall framework across locations, hazard types, and resilience intervention types and to demonstrate the power of narrative exposition to communicate the significance of co-benefits. Two additional narratives remain practical but explicitly hypothetical (i.e., not yet implemented) for illustrative purposes. Section 5 provides the narrative vignettes and associated discussion of co-benefits that fit within the proposed framework of dividends and windfalls.

### 3.2. Co-Benefit, Ancillary Benefit, or Externality?

Fung and Helgeson [11] find that definitions used to describe co-benefits tend to fall, loosely, into three classes: objective, intent, and externality based. Co-benefits of resilience planning may accrue to first or third parties to the investments (e.g., [40,41]). Yet, externalities are fundamentally defined by their associated costs (or benefits) accruing to third parties. Markandya et al. [42] treat co-benefits, ancillary benefits, and spillover effects as synonymous. Similarly, Herrero et al. [40] treats co-benefits, ancillary benefits, and “forgotten benefits” as interchangeable. Ancillary benefits are often used interchangeably with the term co-benefits; however, Mechler et al. [43] draw a distinction between ancillary benefits as those that arise without deliberate planning to meet a non-primary objective.

Aligned with the US Office of Management and Budget (OMB) [44], we take the view that “co-benefits are additional benefits that result from resilience investments that are not directly related to the objective being considered in planning, but are linked to other community-level objectives.” An example of a co-benefit of a resilience measure is a new technology that could result in an increase in resilience and/or reduction in emissions. Within a benefit–cost analysis, how one defines the objectives has a direct impact on what may be considered a co-benefit. For example, an analysis defined around environmental and health objectives would count multiple benefits categories within the main benefits section, rather than listing them as co-benefits. This formulation is synergistic with the concept of additionality, an increase in the quantity or quality of the expected outcomes need to be demonstrable beyond what would have otherwise occurred [45].

### 3.3. Co-Benefits: Addressing Uncertainty or Wishful Thinking?

In studies of the effect of disaster events, there is rarely consensus surrounding the distribution of exposure, vulnerability or possible outcomes [46]. Generally agreed-upon probability distributions are not always available for hazard effects, especially those related to social impacts, and stakeholders differ in their degree of risk tolerance. Natural disaster resilience is complicated by the deep uncertainty surrounding covariate shocks, in particular, and the nuances that affect idiosyncratic disaster impacts.

Temporal uncertainties generally arise from unpredictability of the interaction between outcomes of current resilience efforts and future hazard events. Uncertainty may include the frequency and intensities of future hazards as well as future resources, including human, social, produced, natural, and financial resources. As complex events increase in frequency and impact, the need to understand evolving option values is critical. Challenges also arise from uncertainty in valuing avoided costs of prevented or reduced damage in the aftermath of an event because most cost estimates occur well ahead of an event and ex post evaluation that goes beyond assessing insurable entities is rare (e.g., [47]). In some cases, modeling can address this issue by considering the counterfactual [48] and accounting for co-benefits (e.g., [10]).

Uncertainty surrounding occurrence frequency, magnitude, and timing of a disaster can make it challenging for a community to implement resilience measures when there is a preference to spend a limited budget on capital investments expected to produce certain outcomes in the business-as-usual case. There is a growing understanding that building resilience on a community scale creates benefits in two dimensions: (1) enabling individuals, communities, and organizations to withstand and recover from an objective disruption more effectively and (2) enabling improvement to current systems (i.e., business-asusual/status quo situation) [15]. Furthermore, by lessening the impact of chronic stresses (e.g., crime, poverty, unemployment) through the outlay of small levels of resources over time, a community may be able to better withstand and recover from unexpected disaster events. For example, by reducing poverty and unemployment, a community cultivates the option to redeploy the resources allocated to those objectives in some other manner.

### 3.4. Option Values

Communities must improve their capacity to design and implement sustainable solutions to the problems posed by climate change by cultivating their resilience to anticipated disaster impacts and being mindful of the full value of their opportunities. Dixit and Pindyck [49] describe an important axiom for capital investment; that “opportunities are *options*-rights, but not obligations to take some action in the future”. Options have value. Investment opportunities may be exercised, trading the value of the option for the value of the investment. An investment is generally evaluated by estimation of its net present value, which equals the discounted stream of net benefits derived from a given expenditure. When investment options are exercised, the actions of a community or firm will create new opportunities, and thus, new options. The opportunities beyond the investment horizon are fundamentally difficult to estimate with precision, but to ignore the value of these emergent options is to neglect a most-potent justification for initial action: the benefits of learning and the value of the view from the top of the next hill.

Perfect anticipation of the threats that will face a community is an ideal, but improbable expectation. Instead, communities should seek to identify those opportunities for action that maximize net present value inclusive of the value derived from emergent options. Communities do not need to adopt a complex approach to valuation and can learn from the business literature on incorporating options into investment planning [50,51]. Employing the analogy of a tomato garden, Luehrman points out that with time, some options will ripen while others may rot on the vine or fail to ripen before the end of the growing season. Continual evaluation and selective cultivation of a community’s options for resilience may help to resolve some uncertainty present in the initial planning phase.

Strategies that retain flexibility and avoid unnecessary complexity and cost will support the value of follow-on options. Large expenditures may be necessary, but may also limit follow-on options more than solutions to community problems that leave more public resources for other problems, present and forthcoming. In other circumstances, failing to invest in resilience may result in a community’s decline, and diminished resources for future action. While not a groundbreaking statement, opportunity cost remains ignored at one’s own peril.

Options for follow-on action may form part of the dividends generated by resilience investments. The presence of certain options may prevent a community leader from having to deploy resources in order to secure added operational flexibility. One example is that of a mutual assistance group of fire-fighting agencies and its role in helping communities to secure the resources needed should a large wildfire approach the urban interface. A mutual assistance agreement might entail the costs of coming to an initial agreement, occasionally revisiting the terms, and maintaining a communications channel. The cost of maintaining the option to call in additional help from a neighboring community is small compared to the cost of standing up and operating additional fire-fighting resources that may have a very low-capacity utilization on most days. A dedicated channel for sharing insights into the greatest challenges facing communities in a given region may pay dividends in lessons learned from peer experiences. Strategies that give decision makers additional choices when facing substantial problems increase the likelihood that an acceptable solution will be available. Such an option for additional help constitutes one form of the resilience dividend. In time, communities may find that threats not rising to the level imagined where they require mutual assistance are nevertheless handled better when coordinating operations with one’s neighbors, especially when challenges occur at the interface between communities.

## 4. Classifying Co-Benefits: Resilience Dividends and Resilience Windfalls

This section formalizes categories of co-benefits that contribute to net resilience dividends and net resilience windfalls. As noted previously, ex ante investments in resilience are generally allocated between mitigation and adaptation efforts. Resilience dividends largely align with co-benefits of mitigation strategy investments. Resilience windfalls are largely associated with adaptive capacity that proves especially valuable down the road when the objective event or another event type (e.g., shock or stress) emerges.

### 4.1. Resilience Dividends: A Category of Co-Benefits

Options for follow-on action may form part of the dividends generated by resilience investments. The presence of certain options may prevent a community leader from having to deploy resources in order to secure added operational flexibility. One example is that of a mutual assistance group of fire-fighting agencies and its role in helping communities to secure.

In the context of resilience planning, the resilience dividend has been defined by Fung and Helgeson [11] as “the net benefits from investing in enhanced resilience in the absence of a disruptive event… which captures the intentional or unintentional pursuit of multiple objectives, and the possibility of creating externalities in the process.” Under this definition, planning for resilience can incur co-benefits that address sustainability goals and vice versa (i.e., resilience co-benefits that arise from sustainability planning).

The concept of the resilience dividend helps communities compare investment options using a concept that encompasses multiple objectives and recognizes strengthening the community in the day-to-day, even when a hazard event has not occurred. The resilience dividend can be seen in both the short-term and long-term benefits that span a broad range of benefit types.

Evoking the concept of a dividend, co-benefits that clearly yield significant and tangible benefits help communities view resilience investments as more than a gamble that only pays off in the case of a disaster. The Triple Resilience Dividend Framework [8] presents three types of resilience dividends:

1. Reduced and avoided losses to lives, livelihoods, and assets (1st dividend);
2. Increased development potential by stimulating forward-looking planning, long-term capital investments, and advanced entrepreneurship (2nd dividend);
3. Garnering wider social and environmental co-benefits (3rd dividend).

The first resilience dividend category includes co-benefits when a disaster occurs, while the second and third categories are relevant regardless of the occurrence of the objective disaster event(s). Existing methods of appraising disaster risk management and adaptation investments undervalue the significant societal and economic benefits from taking action early rather than later. Hence, decision-makers view these investments as a gamble that only pays off in the event of a disaster. However, there is increasing evidence that building resilience yields significant and tangible benefits, even if a disaster does not happen for many years or not at all within the timeframe considered.

### 4.2. Resilience Windfalls: A Category of Co-Benefits

The possibility of extremely costly outcomes with small, but positive probabilities can have large impacts on evaluations by benefit–cost analysis related to resilience. These low-probability high-consequence events have motivated a focus on the tail of the distribution of outcomes. For example, a small chance of a truly unacceptable outcome may have a significant impact when evaluating the expected benefits and costs of a resilience project. Thus, communities often take a myopic view of chronic stressors that can culminate in larger disaster impacts (e.g., [52]). To this point, resilience windfalls often are not recognized ex ante due to the unexpected nature of windfalls. We present two sources of resilience windfalls:

1. Investments that lead to a distinct additional performance improvement when the system is confronted by the disruptive objective event (1st windfall);
2. Investments that boost the system’s ability to address the onset of additional disaster events (i.e., shocks), opposed to improving (only) day-to-day operations (stressors) (2nd windfall).

Windfall benefits are large, unexpected gains resulting from unlikely circumstances being realized. Resilience windfalls are typically unexpected during ex ante planning and may be associated with high levels of uncertainty. The first resilience windfall category is akin to the first resilience dividend category, but is nuanced to include the least likely additional performance improvements and often will be only recognized ex post. Furthermore, using multi-objective planning may make the first resilience windfall category less relevant for some interventions. However, funding assistance and sources are often focused upon a single objective and multi-objective planning can be inadvertently discouraged (e.g., [53]). In such cases, identifying resilience windfalls has the potential to expand the objectives relevant in funding assistance applications. Furthermore, ex post evaluation may become more prevalent as we recognize resilience windfalls and resilience dividends that are only relevant after an intervention has partially or entirely taken place.

Another way to think about a resilience windfall is as the discrepancy between expected and actual avoided losses. In this manner, resilience windfalls constitute “sales leads” on opportunities to refine the resilience strategies employed by a community. Resilience windfalls can signal the presence of underexploited opportunities to improve systems performance. In other words, resilience windfalls are the emergent nested real options for resilience that arise out of previous efforts to secure resilience dividends. Initial investments can be designed not only to reap expected dividends, but to position a community to capture good fortune when possible and mitigate the chances for negative surprises. Communities often face asymmetric losses when designing resilience interventions. While a “gold-plated” solution can cost too much, an insufficient investment may prove inadequate to protect a community. If reality sometimes exceeds expectations, we find this possibility less offensive when focused on benefits than costs. Understanding how windfalls emerge over time from resilience investments is critical to a community’s ability to strike a balance between the extremes of investment and insufficiency.

**Figure 1.**
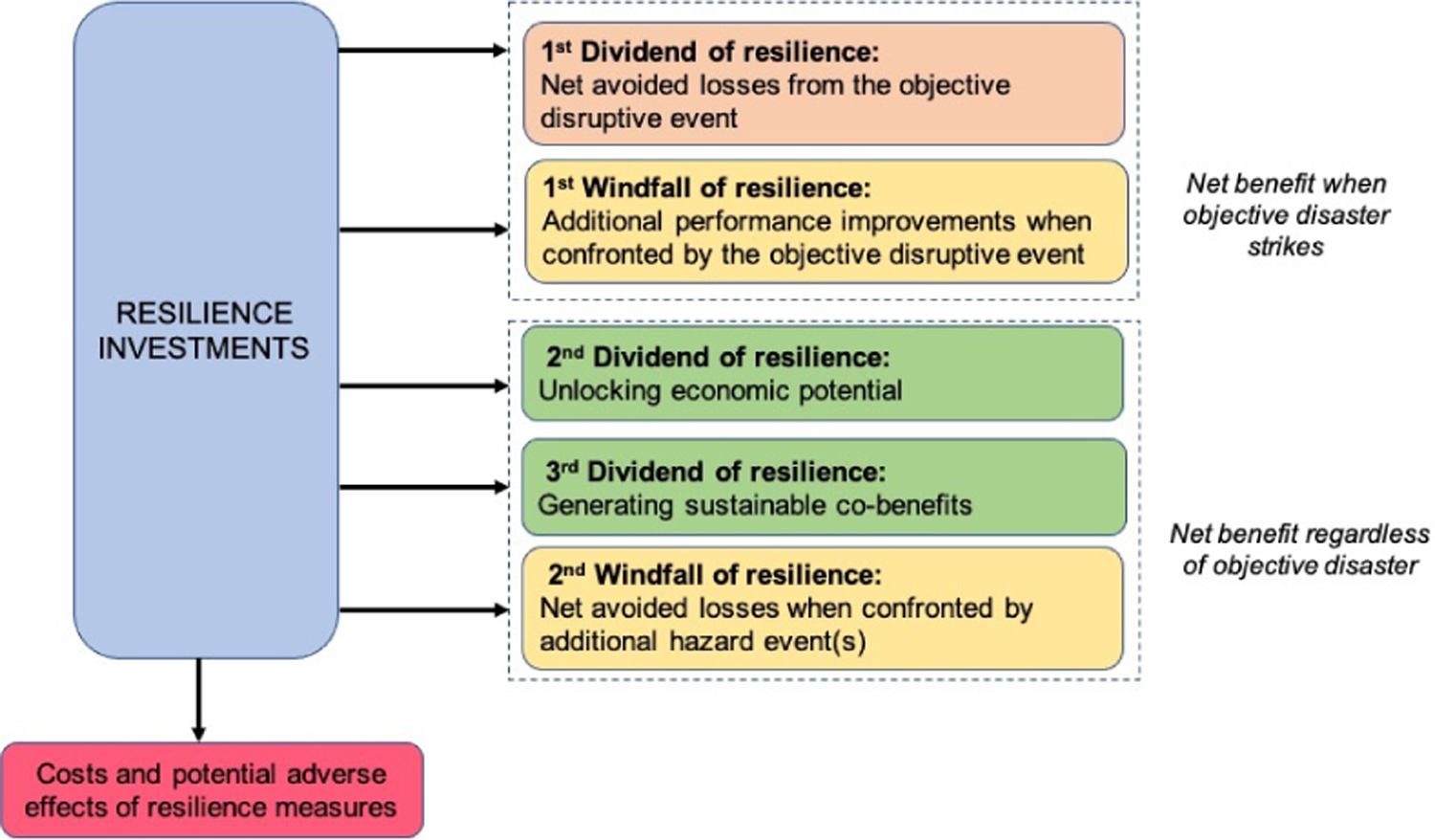
Categories of resilience dividends and windfalls.

A mapping of resilience dividend and resilience windfall categories is provided in Figure 1.

In resilience planning, there is often a narrative component that helps motivate fund-In resilience planning, there is often a narrative component that helps motivate funding and other support for a planned intervention. The value of narratives that provideing and other support for a planned intervention. The value of narratives that provide archetypes is increasingly recognized as an important tool both to manage and understandarchetypes is increasingly recognized as an important tool both to manage and underresilience planning and outcomes (e.g., [stand resilience planning and outcomes (e.g., [36]). Management literature is only begin-36]). Management literature is only beginning to develop a framework for evaluating the quality and legitimacy of narratives. Co-benefitsning to develop a framework for evaluating the quality and legitimacy of narratives. Cospan a broad range of topics and are highly influenced by deep uncertainty; thus, categories of co-benefits are useful in development of resilience planning narratives that are compelling. This is part of what makes them useful and engaging tools to allow transfer across spatial and temporal dimensions.

It is precisely the unanticipated nature of windfalls and a penchant for unwise allocation of resources suddenly and unexpectedly acquired (or enjoyed in the case of the intangible) that commends their inclusion in our planning efforts. Resilience windfalls often accrue as avoided losses and may be non-monetary in nature.

### 4.3. Return on Investment (ROI)

**Figure 2.**
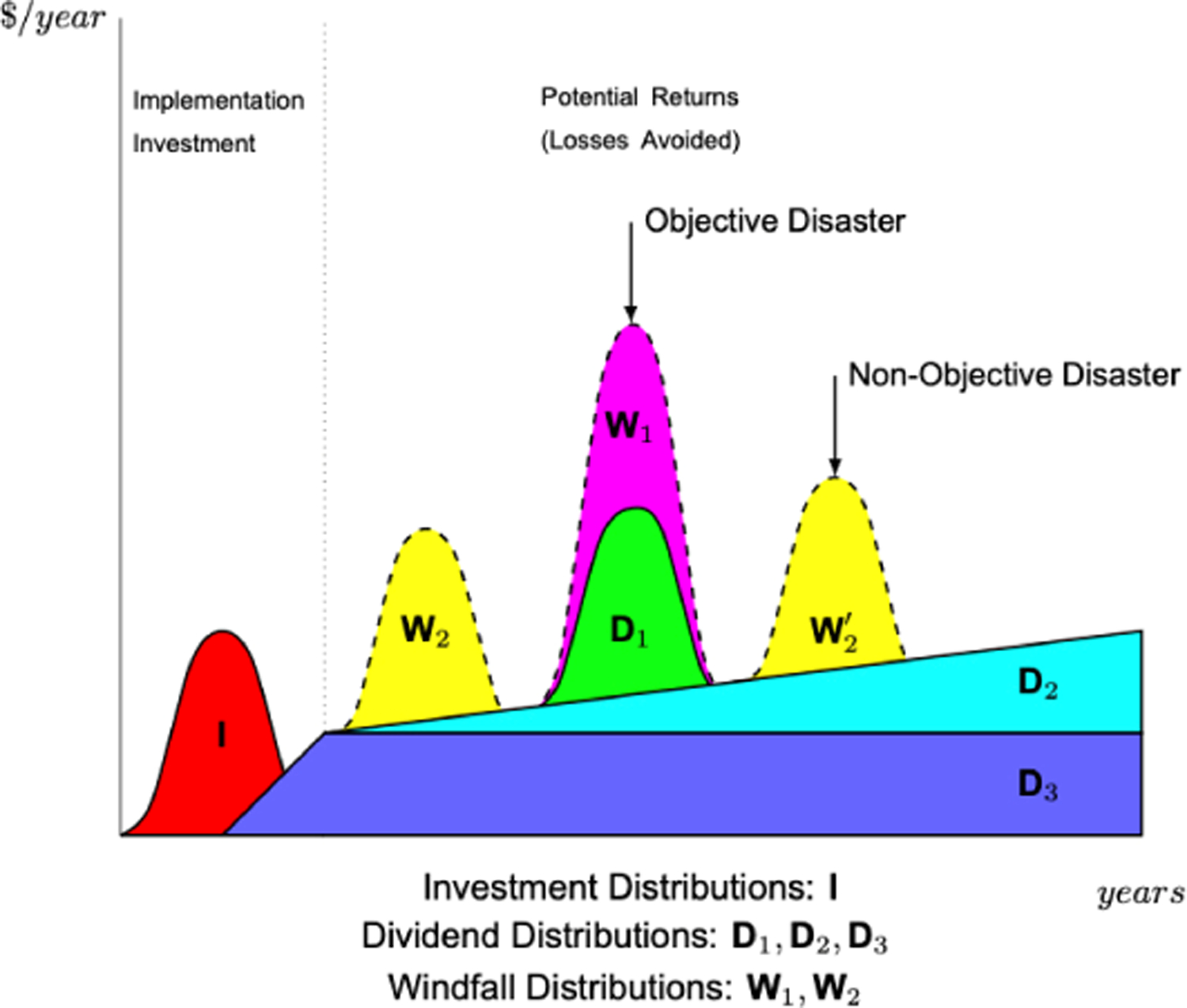
Stylized visualization of possible value streams relevant to ROI estimation incorporating expected values for resilience dividends and windfalls.

**Figure 3.**
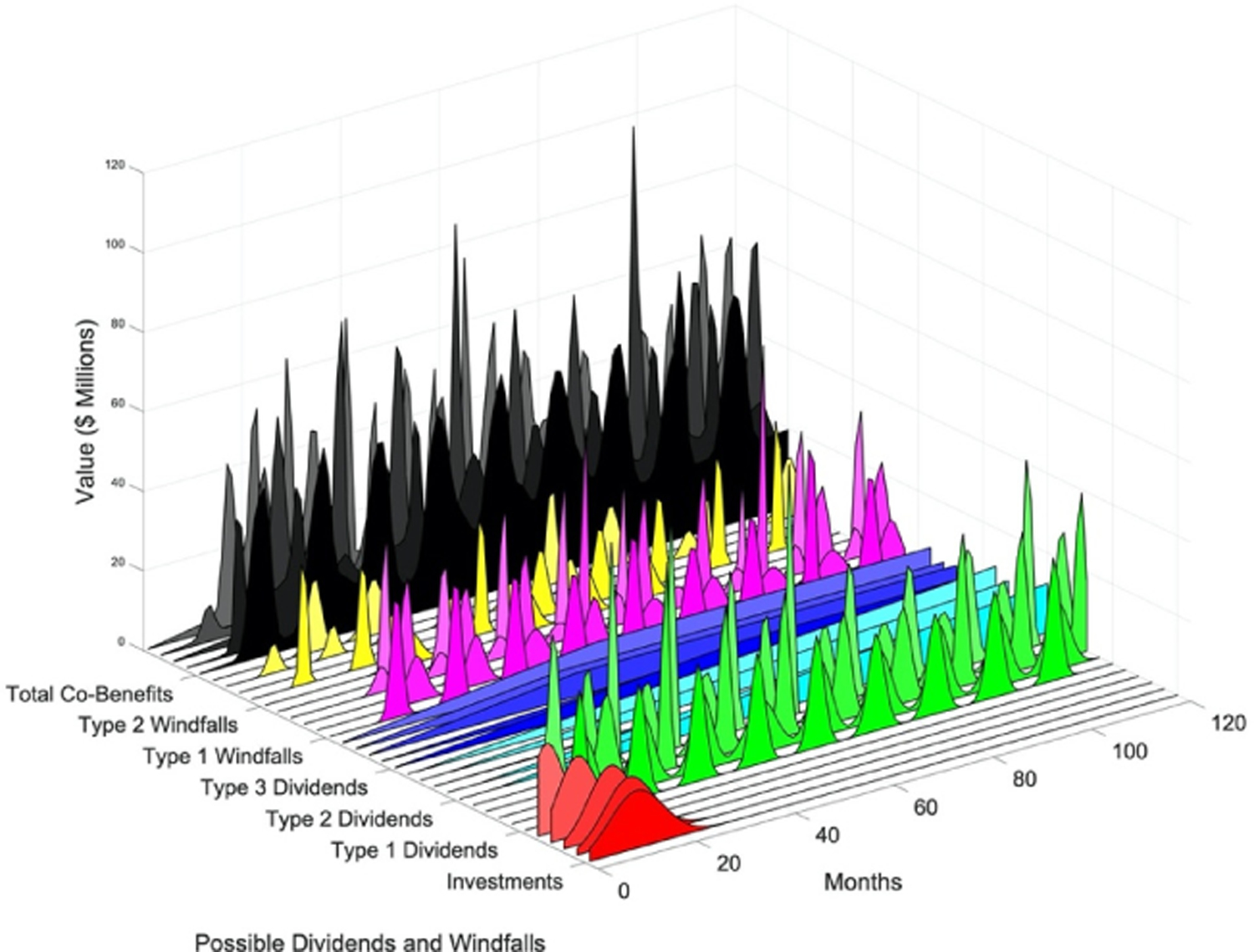
Stylized visualization of possible value streams relevant to ROI estimation including uncertainty surrounding expected value and temporal onset for resilience dividends and windfalls.

Trying to predict a payback period for a resilience project is typically critical to obtaining support and funding for the project; however, determining a meaningful return-on-investment (ROI) may be challenging [54]. Prediction of a payback period compounds magnitude and frequency of expected interruptions, increasing uncertainty. Figure 2 provides a stylized schematic of expected potential magnitude of cost impact in addition to average expected returns from dividends and windfalls—when they exist—throughout a project’s assessed timeline (and beyond). Figure 3 provides additional elaboration on the fact that predictions would be required for both potential magnitude of the (avoided) cost impact, but also of the frequency of occurrence, or alternatively the probability of occurrence within a particular period. Several different realizations of each category of co-benefits streams are presented in Figure 3 to illustrate the uncertainty confronting estimates of ROI. Incorporating expected co-benefits in a non-disaster ROI value [55] can produce a favorable ROI within an acceptable project timeframe.

The ROI is one metric that communities may use to choose between possible projects and to aid in the decision to select a given resilience intervention. ROI estimates and other methods for decision support may benefit from a better understanding and integration into implementation of the co-benefit categories. ROI analysis has expanded to include social and environmental benefits (i.e., social return on investment) that allows for the flexibility to include identified co-benefits. Co-benefits are relevant to the process of arriving at sound conclusions, regardless of the stakeholder’s (e.g., the community’s) preferred assessment metric(s).

Political leaders are expected to make decisions based on expectations formed from the best information available, but their constituents will judge them by the relative resonance of the narratives that describe those decisions favorably or otherwise, especially after the occurrence of a disaster event. That which resonates most might be the dividends and windfalls experienced by community members, and researchers should not shy away from evaluating these experiences empirically. The alignment of narrative discourse with empirical evidence remains crucial to the efficacy of public strategy with respect to sustainable resilience, especially when a number of co-benefits remain difficult to capture fully through quantification (i.e., monetization) alone.

### 4.4. Narrative Expressions of Resilience Dividends and Windfalls

Resilience dividends and windfalls provide a framework for evaluation of resilience planning alternatives that can give value to the options approach. Successful deconfliction of resilience and sustainability goals requires making both concepts inherent to the design and evaluation of prospective strategies. Waiting to incorporate sustainability or resilience into a strategy until a later stage of development will generally entail greater costs borne of the complexity that grows naturally from the pursuit of ad hoc solutions to emergent problems. Maintaining at least an option value for additional sustainability and resilience capacity requires looking forward and learning from other such projects. There are temporal challenges, as noted previously. Identifying some co-benefits may only be apparent ex post a resilience intervention. Thus, ongoing and ex post evaluations are important for identification of such co-benefits and incorporating lessons learned into maintenance of the project at-hand and future interventions. Novel technologies, new institutions, increased organizational variation, or greater structural differentiation all lead to greater complexity in systems and therefore the costs of their employment in solving the problems that confront communities. The cost elasticity of complexity varies from solution to solution, and the effective design process will recognize opportunities for improved efficiency.

The centrality of emotional appeals in marketing materials has long been understood by advertisers [56,57]. Narratives that evoke awe, anger, or anxiety are also more likely to be shared [38]. While we think community leaders ought to assiduously avoid appeals to anger and anxiety, the awe that the experience of windfalls may invoke could be of significant value to the propagation of good ideas across communities. Such a focus is supported by the finding that positive content tends to be more viral than negative content in a review of newspaper articles [39]. Practical value and interest were also major determinants of the likelihood of a newspaper story being shared [39]. Dividends encapsulate the practical value of investments in resilience, and relatedly, drive interest by folks who want to know how they too may benefit from new information regarding the experience of others.

We note that dividends and windfalls constitute two phenomena that are likely to contribute to the virality of the narratives that emerge from community resilience investments. First, while not strictly required, dividends and windfalls are generally positive in nature. The regular accrual of benefits in the form of dividends is likely to engender and sustain positive views of the investments made, even in the absence of the focal disruptive event. Dividends will account for a substantial part of why a given narrative resonates with others because their regularity makes them more salient to the observer. Furthermore, later adoption by other communities should lead to dividends that are relatable to the experience of the originating community. For example, two coastal towns may make similar investments towards resilience against flooding. While the objective event may hit only one of the two towns, both could share the experience of dividends accruing regularly and contribute to the shared narrative of the value of investment. On the other hand, windfalls may contribute to the awe and interest of observers, thus why a narrative is worth sharing between members of the acting and neighboring communities.

As dividends and windfalls may be valued empirically, and with a rigor sufficient to uncover fact, the inclusion of these concepts in the stories we tell each other also ensures that the narratives contain a core of truth. When conveying the findings of research on community resilience and sustainability, we note that “logical-scientific and narrative communication are not just contrasting formats of communication, but represent two distinct cognitive pathways of comprehension” [35]. Dividends and windfalls can be harnessed as examples of logical-scientific communication to be packaged into the broader narratives society already promotes. The emotional content of narratives will continue to be a prime mover behind the proclivity for community members to share their experiences and those of others. However, the inclusion of positive empirical components can contribute to both the scientific quality of shared narratives and the propensity for communities to learn about the opportunities of merit pursued successfully by their neighbors.

## 5. Discussion: Narrative Examples of Resilience Dividends and Windfalls

To demonstrate the importance of incorporating resilience dividends, windfalls, and the associated option values into consideration of community investment options to address resilience and sustainability goals, we offer an overview of a number of resilience interventions in the form of narrative vignettes. The identified resilience dividends and windfalls are noted in Table 1.

Five narrative vignettes are developed and analyzed to determine relevant resilience dividends and windfalls. These illustrative narrative vignettes are presented to test the proposed resilience dividend and windfall framework across locations, hazard types, and resilience intervention types and to demonstrate the power of narrative exposition to communicate the significance of co-benefits. Details of these resilience intervention projects were largely garnered through secondary data sources. Each narrative vignette is presented in three subsections: (1) Situation, (2) Resilience Intervention, and (3) Resilience Dividend and Resilience Windfall. The selected resilience intervention types and communities are as follows: (1) tsunami evacuation (Port Orford, Oregon), (2) air purification (Portland, Oregon), (3) retreat and relocate (Newtok Village, Alaska), (4) rockfall mitigation (Tutuila, American Samoa), and (5) Smart Power Electronics for Wind Farm (hypothetical, high plains). This set of narratives provides a diverse set of project and community types, which can be expanded in future application of this framework.

### 5.1. Port Orford, Oregon Example

#### 5.1.1. Situation

The long tails of the hazard distributions are by definition difficult to counter. Small communities can face the prospect of hazards so immense as to dwarf the local resources they can bring to bear in response. Furthermore, calling for massive expenditures to counter devastating but rare events may fall on the disinterested ears of stakeholder groups that have a hard enough time funding the public services that are more salient and germane to everyday life.

Port Orford is an example of a small community with the potential for a low probability, high impact event. The town, located on the southern Oregon coast, recorded a population of 1133 in the 2010 Census. All coastal communities are aware of the hazards posed by proximity to the Ocean, and in Oregon, the possibility of experiencing “the big one” is a latent threat. With the Cascadia Subduction Zone (CSZ) only 30 miles off the coast of Curry county, Oregon. By one estimate, given a 9.1 magnitude earthquake, this leaves only 10 min from the start of shaking for residents to evacuate before the first waves of the resulting tsunami reach the beach in Port Orford [58]. There are parts of Port Orford where the estimated speed evacuees must maintain to beat the wave (BTW) exceed 10 mph, and the label “unlikely to survive” is applied. The local community needs effective strategies to reduce the likelihood of loss of life in the event of a CSZ earthquake and attendant tsunami.

#### 5.1.2. Resilience Intervention

Port Orford offers an example of a small community with limited resources, that faces the small, but persistent risk of a catastrophic event. Hard solutions to protecting the community, such as massive sea walls, or relocating every house in the hazard zone are unlikely to be economic in any sense of the word. However, some creativity in working the problem can go a long way where limited resources cannot.

First, if rendering local buildings maximally resilient to a tsunami is untenable, improving the viability of evacuation routes seems to be a more approachable objective, at least initially. An analysis of the prospects for Port Orford compares the evacuation routes and necessary travel times with and without the presence of hypothetical trails allowing residents of low-lying areas to more-swiftly reach the headlands where they could escape incoming tsunamis [58].

While investments in trails may seem a low-tech solution to the evacuation problem, and potentially vulnerable to disruption, they are also relatively inexpensive and aligned with best practices. Current guidance encourages people to “go on foot” if they can when evacuating as roads may be damaged in ways precluding vehicular travel. Alternatively, stronger bridges constitute a clear example of an investment that might help avoid a catastrophic loss in a major earthquake that would otherwise have leveled a less resilient conveyance, but the most important value proposition to the community connected by such a bridge is in what it can do for the community every day under normal operating conditions.

#### 5.1.3. Resilience Dividend and Resilience Windfall

The hypothetical paths do not solve all the problems of evacuation and may suffer damage during any earthquake that causes a tsunami. However, if thoughtfully designed, such trails might serve an everyday purpose for which their existence can be justified economically, namely recreation and improved connectivity of the local transportation network. Daily foot traffic on these new trails would constitute revealed preference for the additional amenities offered by the expansion and interconnection of existing transportation networks. Through regular use, such infrastructure investments can pay dividends. In the event of a major CSZ earthquake, another potential route to safety is available at the critical hour, helping to avoid or reduce the loss of human life. If the additional routes relax congestion on other avenues of egress during a tsunami, the discrete boost in performance experienced by the rest of the transportation network could classify as a resilience windfall.

Closer inspection of the hypothetical high ground trails in Port Orford shows that these additions to the trail network are not interconnected with other trails and roads atop the headlands [58]. Therefore, as presently hypothesized, the resilience dividends associated with the amenity values of the trails are limited to perhaps the value of a new scenic viewpoint. Nevertheless, once constructed, such a scenic viewpoint would confer upon the community the option to connect it with other trail or road networks. Compared with more expensive hard solutions to the problem of evacuation feasibility, building escape trails likely permits the community to keep more resources in reserve to meet other emergent challenges. Community action on evacuation feasibility also sends an important signal to residents that local threats are being taken seriously and that accessible and practical strategies are being pursued. Through inaction, the community could miss out on the resilience dividends planning efforts can confirm upon local economic development. All else being equal, investment and business prospects in communities actively planning for threats is likely greater than for peer communities relying on wishful thinking.

### 5.2. Through the Fire—Portland, Oregon (and Adjacent Communities)

#### 5.2.1. Situation

On Monday, 7 September 2020, Labor Day, forest fire smoke descended over Portland, Oregon. Like many cities on the west coast, the cumulative airborne detritus from millions of burned acres blocked the sun, cast an orange hue across the sky, and threatened to harm the respiratory health of residents not protected by adequate building envelopes, and air filtration systems. The smoke from myriad fires had a chilling effect on outdoor activity within the city. Nearby, residents of the wildland urban interface (WUI) to the south and west of Portland faced evacuation orders and the prospect of losing home and livelihood to a fire season beyond comparison in living memory.

Those who evacuated had to seek safety with friends, family, at hotels, or at public shelter facilities. What might have proven a logistical or financial challenge any other year presented a new set of threats during a pandemic when close living conditions presented other health risks. Residents of outlying communities that had to abandon one of the most-effective strategies for social distancing, staying at home, often had to take shelter in relatively greater proximity to others. Infectious disease such as influenza can be a threat in shelter facilities, and the health and safety of vulnerable populations forced to avail themselves of such resources is rightly a concern of shelter managers. This is part of why it is generally preferable that people be able to remain in their homes unless local hazards mandate their relocation. During a pandemic, there is increased value in resilient infrastructure and effective public service organizations (e.g., first responders, emergency managers, fire-wise community groups) that enable people to stay in their homes, or wherever they can best meet their own set of needs. The greater the set of concurrent hazards that threaten a community, the greater the value of infrastructure resilience to those hazards.

The fire did not directly threaten the city center; however, hazardous air quality meant that the relative value of amenities internal to one’s home increased and the relative amenity value of the park down the street decreased. Furthermore, the value of environmental services provided by home heating, ventilation, and air conditioning (HVAC) systems and plug-in air filtration devices increases with the concentration of PM2.5 outside and the risks of contagion presented by local caseloads.

The wildland fire threats faced by communities within and in proximity to Portland, far from being unique, were present in towns across and beyond the American west. By one estimate [59], 100 million Americans live in the wildland-urban interface (WUI). While dangerous air quality from wildland fire is an indiscriminate adversary of public health, its effects are often concentrated among those communities with the fewest resources to mitigate the problem. Left unaddressed, air pollution from wildfires will continue to threaten the quality of life enjoyed by all affected communities.

Contingency planning by infrastructure operators often encompasses a wide variety of potential threats to reliable operation. However, scenario’s entailing overlapping hazards are generally considered to be of low probability and are difficult to anticipate over a typical planning horizon. In spring of 2020, utilities along the gulf coast and southeastern seaboard certainly knew they would need to be prepared to respond to a hurricane with strategies that accounted for the added challenges presented by a global pandemic. At the same time, utilities in western states knew that their preparations for the upcoming fire season could not ignore the realities of operating during a pandemic. However, six months prior to the onset of contagion, contingency planners did not know how dramatically their operating environment would change.

#### 5.2.2. Resilience Intervention

Affordable appliances exist for filtering indoor air pollution from pets, pollen, cooking, and other sources. Certainly, plug in appliances for improving indoor air quality and replacement air filters for HVAC systems are relatively less expensive than upgrades to building envelopes and air handling systems. Many households report purchasing air filtration devices to contend with latent irritants such as pet dander or pollen. However, the environmental services provided by these technologies scale with the set and severity of irritants incident on the household, and consumers may enjoy a resilience windfall as the appliances that serve them every day under normal conditions prove even more valuable when air quality deteriorates.

That we can address a problem as large and sinister as widespread hazardous air quality with off-the-shelf, consumer-grade, technical solutions is laudable, but only if the infrastructure on which such solutions rely is itself resilient. Should the electric grid maintain its track record for reliability and resilience to a diverse set of hazards, industry will have a strong incentive to develop technical solutions that solve problems with electricity. As more solutions depend existentially on the operations of the electric grid, the value of resilient electricity infrastructure continues to rise. Members of outlying communities that lost power, either because or as a precaution against the fire, numbered in the tens of thousands, and now know the value of resilience all too well.

Knowing is a crucial starting point, but deconfliction of the strategies that are typically brought to bear against overlapping hazards may nevertheless prove untenable. De-energizing transmission lines to mitigate against the ignition of forest fires may mean that residents reliant on electricity for life-supporting appliances such as air conditioners, or air filtration, continuous positive airway pressure (CPAP) machines, or even oxygen concentrators must adopt new strategies for dealing with both underlying conditions and smoke from other fires in the region (even households with onsite backup generators may have fuel requirements that cannot be met practically when infrastructure interruptions hit gas stations). Such new strategies may be limited to evacuation. It is worth noting that even if equipment can be successfully evacuated with people, there is no guarantee that shelters will have adequate facilities to host the appliances needed by evacuees from vulnerable populations. As a consequence, health care facilities may face additional demands on their limited resources to help people who would not seek such help if not for having to evacuate. For those households that escape conflagration directly, the interruption of infrastructure services, especially power and water, may be the determining factor with respect to whether a family is able to stay in, or return to, its home.

#### 5.2.3. Resilience Dividend and Resilience Windfall

Anticipating all the complexities that overlapping hazards present is likely beyond even the most sophisticated actors, but that these complexities embody operational costs to families, infrastructure systems, and the broader economy is clear. Effective strategies for strengthening the resilience of infrastructure are of greater value when hazards abound, and few alternatives exist. It is precisely during these times, when beset by hazards on all sides, that we are likely to observe the resilience windfall manifest as additional avoided losses due to the worsening conditions associated with infrastructure interruptions and the rising value of new opportunities to maintain continuity of operations.

Investments in the resilience of the electric grid prove especially valuable when they enable people to stay in their homes and avoid initiating a cascade of additional problems for them and community resources to solve. The investments made by families to improve the indoor air quality and alleviate some allergy related suffering may pay dividends each year when fire season occurs. At a time when a pandemic further reduces one’s options, investments in improved indoor air quality may deliver windfalls that transcend the originally investing party. For example, the family that retains power can use its electricity-fueled air filtering devices and stay at home. This family is thus able to maintain its pandemic safety posture, avoid exposing itself and others in the community, save community shelter and recovery resources for those less fortunate, and reduce the workload and exposure of first responders. The complexity of responding to wildfire in a pandemic leads to both the threat of additional injury to communities and the opportunity to realize co-benefits elevated in scale by the very challenge of the moment.

### 5.3. Relocation of Newtok Village, Alaska Example

#### 5.3.1. Situation

Progressive shoreline erosion along the Ninglick River, combined with permafrost degradation and seasonal storm flooding, began to seriously threaten the land on Nelson Island that the Village of Newtok, Alaska. The Native Village of Newtok is a federally recognized Tribe of Yup’ik Eskimos who live along the Ninglick River on Alaska’s Bering Sea coast.

Lack of permafrost in the area and projected climate change scenarios yielded no cost-effective way for the village to stay in its current location; in 1995 the community originally decided to pursue a move of location. A collaborative planning group was established by the Village in 2006 to determine how to relocate to higher ground, especially in sight of coastal storms that were becoming more frequent. In the period of 2002–2017 there were six federal disasters declared involving Newtok due to specific flood events. By 2017, Newtok had already lost its sewage disposal system, solid waste site, barge landing, and boat dock as a result of coastal storms and erosion. Furthermore, the loss of the community’s boat dock and barge landing site in 2005 created significant safety issues. Residents were starting to experience significant public health issues with no way to properly handle human or other solid waste. The US Government Accountability Office Report 04–142 [60] identified Newtok as one of four Alaska villages in imminent danger that need to relocate to a safe site.

The residents of Newtok wanted to preserve their identity as an independent village within the region, as their tribe has lived in the area for over 2000 years. The per capita median income of the residents was estimated at 68% below the national average; however, the community and culture continued to survive under a subsistence economy.

#### 5.3.2. Resilience Intervention

The Village of Newtok was relocated to a new site nine miles upriver, Mertarvik, with the hopeful Yup’ik meaning of “getting water from the spring.” The Village of Newtok had acquired Mertarvik in 2003 in a land swap agreement with the US Fish and Wildlife Service [60]. The total cost of the relocation project has thus far been split between the state of Alaska and Federal funding sources. Mertarvik construction of basic infrastructure was completed in 2014. Military barracks from Joint Base Elmendorf-Richardson in Anchorage were retrofitted in lieu of new construction; this arrangement is estimated to be more cost-effective than building new houses from scratch, considering Alaska’s high construction costs. The first residents from Newtok moved to Mertarvik in October 2019; a total of 140 people of the Village’s 350 person population relocated as of February 2020.

Development of Mertarvik’s infrastructure system is ongoing; however, current infrastructure is able to address basic socio-economic needs of the community sufficiently while being resilient to coastal storms. Unlike Newtok, Mertarvik is connected by four low volume gravel roadways in addition to the quarry road and the landfill access road. If this community had not moved, it is expected that their previous location would have no longer been viable once the school and airport were condemned.

The case for the relocation project involves a great deal of compound hazards that span acute disaster resilience and long-term climate sustainability. There is no clear state or national policy funding relocation of communities threatened by flooding, erosion, and/or permafrost degradation. Thus, the FEMA Hazard Mitigation Assistance provided largely addressed resilience to the primary natural hazard of coastal flooding with consideration for winter storms. Consequently, there is no dedicated funding stream to complete relocation projects in a manner that does not involve slow, steady payments towards development, opposed to a fast move [61].

#### 5.3.3. Resilience Dividend and Resilience Windfall

The primary objective of this project is to reduce or eliminate potential physical damage to all structures and infrastructure in Newtok from future storms/flooding and to offer the community members associated life-safety benefits. Furthermore, this solution is more effective than alternatives that iteratively repair or strengthen existing buildings or infrastructure in-place.

The overarching resilience dividend which accounts for a large number of co-benefits is that the social fabric of the village (i.e., social and cultural benefits) is maintained and strengthened, opposed to being allowed to slowly decline or eventually vanish completely. The case of this managed retreat to a new piece of land fundamentally challenges the definition of community typically considered in resilience planning. This holistic approach to community relocation ensures that people, property, and tribal resources are more resilient to both natural hazard risk and sustainability against climate change impacts. This project provides a unique approach to acquisitions that embraces managed retreat, rather than the more common single-parcel buyouts that often results in “checker-boarding” and an incomplete increase in resilience capacity.

When interviewed in August 2020, there was the impression that residents of Mertarvik gained more than just solid ground; they report being healthier and living a more traditional Yup’ik lifestyle [62]. The co-benefits for access to healthy food and water is significant. Additionally, the co-benefit of moving into different structures, opposed to retrofitting existing homes in Newtok, many of which had significant molding issues, is that they have improved systems, such as indoor water access fed by Portable Alternative Sanitation System (PASS) water tanks. Furthermore, a new electric system powers streetlights and warms structures, such as homes and the community center.

Though frustrating to the Yup’ik tribe, the slow financing of the construction of Mertarvik with a steady stream of smaller payments, opposed to a project that was fully funded from the start seems to have encouraged exemplary design by piloting different construction methods [63]. In many ways this creates a resilience windfall, as the community is more resilient than expected to disaster events, such as flooding and the COVID-19 pandemic. Furthermore, the staged development of Mertarvik and the resilience of current Yup’ik residents encourages those remaining in Newtok to make the move upstream to the new community location. In September 2019 the play “Before the Land Eroded” was performed by Yup’ik high schoolers during “Water is Life” week facilitated by the National Tribal Water Center (NTWC)—“Newtok. Before the land eroded, I was once there. This river is taking it. The land is sinking, or the water is rising—or both.” These were the opening words of the play which tells a story of environmental change, resilience, and cultural continuity [64].

### 5.4. American Samoa Rockfall Mitigation Example

#### 5.4.1. Situation

Tutuila is the largest island and economic hub of American Samoa; 95% of American Samoans live on the island. It is marked by some mountainous regions with a highest point of 653 m (2142 feet) and is an attractive tourist destination due to beaches, coral reefs, and World War II historical sites. Heavy rain showers can occur all year and persistent flooding often causes damage such as landslides, electrical power failures and road and culvert damage. Tropical cyclones are fairly common in this region, occurring generally once every decade, the most recent was Tropical Cyclone Gita in February 2018.

Earthquakes and tsunamis also occur in the area, the most recent of which took place in September 2009. Following the submarine earthquake of magnitude 8.1, four tsunami waves ranging from 15 to 20 ft (4.6 to 6 m) high, made an impact. The water from these waves flowed inland approximately 100 yards (100 m), leaving automobiles stuck in the mud and some roads impassable. Tutuila is a fairly narrow island, measuring approximately 21 miles (33 km) across. Thus, transportation redundancy is not feasible and furthermore, over 90% of the land is communally owned, making land planning tenets that work elsewhere less relevant in this context.

#### 5.4.2. Resilience Intervention

The 2009 Samoa earthquake and tsunami spurred reassessment of mitigation plans for Tutuila, which addressed landslide remediation. In 2015, the American Samoa Department of Public Works received $3.3 million in FEMA Hazard Mitigation Grant Program (HMGP) funds to implement rockfall mitigation at four locations on Tutuila, where landslides are a persistent problem, especially during extreme disaster events. Landslides along American Samoa Highway 001 (AS001) are a hazard for both pedestrian and vehicle traffic. The project removed loose rocks from the four sites and used wire mesh to stabilize the slopes. “Best management practices were used to ensure pedestrian and vehicular safety, and to protect against adverse impacts to the environment, cultural resources, and air and water quality” [65].

This mitigation activity meets the primary objectives of reduced loss of function of infrastructure systems from large-scale natural disasters and reduced cost for cleanup after a rockfall event. Furthermore, in the more developed areas along ASH 001 the mitigation project prevents direct damage to structures and provides life-safety benefits from reduced injuries and potential death.

#### 5.4.3. Resilience Dividend and Resilience Windfall

Landslides and rockfalls on volcanic islands in tropical climates are characteristic landscape shaping features. To this point, under the business-as-usual case, landslides and rockfalls are continuously shaping tropical islands’ landscapes and contribute to the volcanic geoheritage of Tutuila. Due to the small-size and steep terrain of the island, there are limited natural resources and fresh water available.

AS001 is one of only three highways on the island and it intersects both of the other highways. In addition to staving off impacts of extreme natural hazards, the resilience dividend of this project addresses benefits to reduced nuisance flooding and helps to maintain the natural beauty of the island and health of its water resources. In turn, the tourism industry is free to continue to develop, as confidence of developers and tourists may be increased. Tuna fishing and work in the one remaining tuna processing plant are the mainstay forms of employment in Tutuila outside of tourism and retail, with canned tuna the primary export. This tuna plant lies along AS001, and the highway is the main access to it.

The resilience windfall derives from curtailing necessary cleanup and repair of roads and vehicles in the case of natural disasters outside of earthquakes and tsunamis, such as the case of Tropical Cyclone Gita. During such events, access to this mainstay roadway allows for evacuation and access to healthcare.

### 5.5. Hypothetical Wind Farm Example

#### 5.5.1. Situation

A community has set aggressive renewable portfolio standards for its electric generating stations. A primary objective is to improve sustainability by reducing power plant emissions. Furthermore, the community is aware of the centrality of critical infrastructure such as the electric grid to the economic well-being of its members and wishes to avoid steps that could reduce the resilience of the grid to the hazardous and variable operating environment in which the community lives. Storms have increasingly threatened the grid and constraints on local fuel delivery for thermal generation can bind during periods of peak winter demand. The community is looking for a coherent investment strategy to meet its sustainability and resilience objectives with competitive solutions.

#### 5.5.2. Resilience Intervention

A local electric utility invests in a wind farm to increase the percent of generation coming from renewable sources and reduce dependence on limited fuel delivery systems. The utility decides to design its system using doubly fed induction generators (DFIG) along with other power electronics and maybe storage to maximize the wind farm’s ancillary service capabilities. The wind farm’s ability to support the overall function of the grid is inherent to the facility from the start of the design process through the end of implementation. Once operational, the wind farm supplies power in a temporal profile that follows the diurnal and seasonal patterns of the region, yet retains the capability to modulate operations in support of the grid. With time, operating experience, and forecasting resources, this support capability grows in value to the community. As wind output often peaks at night when demand is low and the fewest conventional generating assets are online to support operations, the advanced power electronics at the wind farm allow it to sell frequency control services to the grid when they are most needed, and thus most valuable. If the wind farm can displace conventional reserves, the system’s sustainability may be further improved.

On a particularly stormy night, a fault occurs on a distant transmission line, causing grid frequency to drop precipitously, only to be arrested by the fast frequency response of the DFIG wind turbines still feeding into the grid. The swift action of the wind resources bought the rest of the grid enough time to marshal its resources for resilient operation. The investment intended to make the grid more sustainable can also make it more resilient.

#### 5.5.3. Resilience Dividend and Resilience Windfall

The first sustainability dividend produced by the investment in the wind farm with advanced power electronics is that of the reduced emissions from fossil fuel generation that is displaced by the lower marginal cost alternative resource. A wind farm composed of turbines employing doubly fed induction generators can supply a resilience dividend through its ability to rapidly ramp power output to provide fast frequency response that previously required maintaining additional conventional generation on standby for contingencies. The accompanying reduction in emissions from conventional reserves supplies a second sustainability dividend. Finally, the DFIG wind turbines could deliver a literal resilience windfall, potentially averting customer outages and associated costs, by supplying fast frequency response services if a power plant outage or downed transmission line threatens to dangerously reduce grid frequency.

### 5.6. Discussion of Example Resilience Dividend and Windfall Narratives

The narrative vignettes highlighted in this section point to the diversity of forms which co-benefits can take across community types and disaster resilience planning contexts.

In coastal towns facing tsunami risks, trails built to offer improved options for evacuation may contribute resilience dividends as the public trails and scenic viewpoints are enjoyed by residents and tourists. The development of evacuation options may unlock some of the local economic potential by signaling to concerned parties a willingness to plan for resilience and mitigate risk.

Household investments in air purifiers, purchased to protect residents against pollen and other common irritants may also make their homes more resilient to smoke from regional wildfires during the fire season. A resilient electric grid remains crucial to unlocking the economic potential of electronic devices designed to solve a host of problems, many of them health related.

Proactive resilience planning efforts that allow small remote villages to retreat from the encroaching sea and endure occasional storms, pays dividends every day the community fabric is maintained at the new location. Economic potential might be squandered if a lack of planning and commitment led to the dissolution of these communities. Likewise, rockfall remediation on tropical islands can pay dividends by mitigating other common challenges such as nuisance flooding and helping to secure critical infrastructure such as road networks.

Neglecting to consider potential resilience dividends and windfalls as core concepts in the design and evaluation of opportunities will also lead to suboptimal allocations as the value of options created by a given strategy with respect to improved sustainability or resilience are likely to go unaccounted. Sustainable strategies for improving resilience avoid unnecessarily large cost structures that limit options for coincident and subsequent investments. Resilient strategies for sustainability must be flexible to remain cost effective in a changing environment. A changing climate may imply rapidly diminishing returns to a given set of strategies for solving a community’s problems.

Programming expected dividends and the potential for windfalls into a community’s strategies to improve resilience helps to sustain the effort over time. The demand that investments be fully self-funding could be onerous, but resilience can be enhanced by reducing the net obligations imposed on society by an investment strategy in sustainable solutions through the lens of resilience dividends and windfalls. Additionally, though communities differ greatly, lessons learned through the exposition of these co-benefits are applicable to similar projects in other communities.

## 6. Summary and Conclusions

To sustain resilient communities, especially as the VUCA state moves us towards increasing complexity, transformative innovations in urban planning, industrial technology, and environmental policy are needed. In particular, addressing these objectives when considering infrastructure systems that support social and economic functions is critical. The three major global climate agreements—the Sendai Framework for Disaster Risk Reduction [66], Paris Climate Agreement [67], and the Sustainable Development Goals [24]—all emphasize the need to integrate disaster resilience and climate risks with continued sustainable development concerns. Thus, inclusion of resilience dividends and resilience windfalls that concurrently generate value for addressing acute disasters and chronic conditions towards sustainability can dramatically change the tenor of resilience planning moving forward.

As goes the old riddle, “A lilypad in a pond doubles in size each day. On which day does it cover half of the pond?” [68]. The answer is only one day before it covers the whole pond. This is a helpful analogy for the need for sustainability planning; everything is manageable until a tipping point is reached. The ability to classify co-benefits as contributions to resilience dividends and resilience windfalls is a critical step to structure thinking about co-benefits across diverse community types in terms of socio-economic factors, shocks and stresses faced, and objectives valued by relevant stakeholders. Communities that are not able or decide not to spend resources on dealing with chronic conditions (e.g., aging infrastructure, a stagnant economy) largely become less resilient. In turn, reliance on external resources and delaying resilience planning until it is clearly needed causes greater social costs in the long-term.

There are resource costs to complexity in resilience planning. It is important to limit cost escalation through aligning (i.e., deconflicting) goals as well as cultivating dividends, potential windfalls, and the option value of opportunities. It is often difficult to ascribe monetary values to co-benefits, especially when there is a mix of co-benefits evaluated within an analysis [69]. A first step in streamlining co-benefit valuation is to strengthen the categorization of co-benefits and to provide narratives that help communities think through co-benefits that may not be typically recognized nor easily valued.

In addition to co-benefits that can arise from resilience planning, such activities can also result in co-costs (e.g., [11,70,71]. For example, the use of community centers for hurricane and tornado response during a pandemic results in exposure to communicable diseases (e.g., [72]). The categorizations of co-benefits presented in this paper do not focus on co-costs; however, these are relevant as part of net co-benefits. Additional consideration of co-costs is left for future work.

Communities involve large groups of sub-groups with varying levels of vulnerability and stakeholder roles related to given resilience planning efforts. To this point, equity in the provision of resilience dividends and resilience windfalls remains an important consideration. This is left for future assessment as additional narrative vignettes of resilience dividends and resilience windfalls are collected. Future work may also evaluate the factor intensity of resilience dividends and windfalls, especially as adding to additional mitigation and adaptive capacities to the community. The use of narrative expressions is relevant in (1) project design towards more inclusiveness of co-benefits and expansion of the number and types of potential project beneficiaries and (2) to project communication to build support for investments in resilience and sustainability.

Evaluation of resilience dividends and windfalls can help justify resilience planning for a given community and the value of considering additional objectives. As a database of such co-benefits is built up, these can be applied to planning processes and learning in other communities facing similar concerns and circumstances (e.g., vulnerabilities). Co-benefits are not guaranteed to accrue in all cases and the resilience dividend and resilience windfall as categories, likewise, may only apply to a subset of resilience interventions. Yet, from the perspective of society at large, there are significant resilience dividends and resilience windfalls that accrue from singular projects, which viewed narrowly may be recognized as positive externalities. The challenge facing humanity is to live sustainably within ecological and physical limits and account for the societal boundaries needed for social cohesion and well-being. Informing a robust discussion around co-benefits and associated categories of resilience dividends and windfalls can help motivate increased learning and cooperation, and effectively advance projects that address both resilience and sustainability. The framework developed herein is applicable to processes, institutions, and policies that provide funding for resilience projects at the local and global levels.

## Figures and Tables

**Table 1.** Community Examples of Resilience Dividends and Windfalls.

	Resilience Intervention	Tsunami Evacuation	Air Purification	Retreat and Relocation	Rockfall Mitigation	Smart Power Electronics for Wind Farm
	Community Example	Port Orford, Oregon	Portland, Oregon and Adjacent Communities	Newtok Village, Alaska	Tutuila, American Samoa	Hypothetical, High Plains
**Resilience Dividends**	**Type 1**	Avoid loss of life through additional tsunami escape routes	Resilience on high pollen concentration days (seasonal)	Ability to cope with storms	Ability to cope with earthquake and tsunami impacts (e.g., emergency evacuation)	Electric grid frequency control services help to avoid outages due to contingencies
	**Type 2**	Public scenic trails and increased productivity and increased quality of life (and greater housing prices)	Allows efficient and uninterrupted work and recreation opportunities	Healthy living conditions allow for increased productivity	Increased tourism sector potential. Maintain and strengthen critical tuna packing sector	Allows wind farm to sell ancillary services in support of grid operations and not just energy
	**Type 3**	—–	Energy efficiency and better health outcomes	Sustainable infrastructure; sustainable “social fabric” and way of life	Maintain landscapes and increased land conservation; Safeguards travel routes needed for first responders to everyday issues (like hospital transport)	Additional operating flexibility to displace conventional generation, reducing electric grid emissions
**Resilience Windfalls**	**Type 1**	Increased diversity of evacuation routes. Reduced congestion on some other paths of egress	—–	Mitigation and adaptation to thawing permafrost, especially seasonal variations, which xacerbates storm impacts	—–	Fast frequency response of find farm with advanced power electronics may out-perform conventional strategies for stability, like inertia
	**Type 2**	Easier evacuation for other disaster events	Greater resilience towards wildfire smoke. Greater ability to stay in housing contributes to preparation for public health emergencies (e.g., COVID-19 pandemic)	—–	Maintain critical roadway and emergency routes during cyclone and nuisance flooding events	Properly equipped wind farm may offer stability services when the rest of the grid has limited ability to do so

## Data Availability

Not applicable.
